# The Nursing Supplement

**Published:** 1890-01-11

**Authors:** 


BEING THE EXTRA NURSING SUPPLEMENT OF "THE HOSPITAL" NEWSPAPER.

All Contributions for this Supplement should be addressed to the Editor, THE HOSPITAL, 139-140, Salisbury Court, Fleet Street, London, E.C., and should have the word "Nursing" plainly written in left-hand top corner of the envelope.

### En Passant.

CHRISTMAS CARDS.—We have once more to thank our kind correspondents for the good wishes and pretty cards which many of them have sent to "Mr. Editor." Once more we repeat that our hearty good wishes are with all nurses throughout the land, and that we most earnestly wish them every success and happiness throughout the present year.

SHORT ITEMS.—At the opening of the Hartley Memorial Wing of Sunderland Infirmary, the nursing sisters were referred to with praise.—Birmingham Workhouse Infirmary has been embellished by paintings done by the nurses, those of Miss Burgess being specially admired. Prince and Princess Edward of Saxe-Weimar visited the Kilmainham Hospital on Christmas Eve and gave presents to the inmates and shawls to the nurses.—Several nurses at Charing Cross and other of the metropolitan hospitals are laid up with influenza.—Miss Mansell commences some excellent "Notes on District Nursing" in this month's *Nursing Notes*.—A general meeting will be held at the Midwive's Institute and Trained Nurses' Club, 15, Buckingham-street, on Friday evening, January 31, at 7 o'clock, when the attendance of members is requested.

NURSES AT MELBOURNE. — Mr. Williams, the secretary of the Melbourne Hospital, who lately visited England, and appreciated our nursing arrangements, seems to be much disgusted with his home welcome at his own hospital. Eight nurses have complained that since Mr. Williams returned from Europe he has treated them severely, and called them by their surnames, instead of addressing them as "Nurse." Here is the evidence of one of the nurses before the committee: "Mary Cootes (two years and a quarter in the hospital) said that the nurses who had tendered their resignations last April were being gradually tantalised out of the hospital. Mr. Williams had complained to her of her arrangements of combs and brushes, soap and flannel, and eggs; and had let her know, through the matron, that she was not a desirable person, and that he would consider whether she should go back to night duty or be got rid of. She thought he wanted to get her from the nursing staff, as he had done to others who had left on his account, and she felt unsettled. The treatment was better before he went away. The food was badly cooked and served, but she had not complained of that to the Visiting Committee. She did not like to be called by her surname." Mr. Williams ought to have observed that if our nursing arrangements are improved, it is because nurses are now under trained matrons. The secretary ought not to be called upon to interfere with or in any way correct the nurses, who ought to be solely responsible to the matron. Mrs. Miller, the matron of the Melbourne Hospital, said there had been great dissatisfaction since Mr. Williams's return. She considered that he pried too much into matters which she thought should be under her control, namely, the management of the female department. The matter of food might be amended. Reforms may be, indeed, we know are, badly needed in nursing circles in Melbourne, but the reforms need not be harshly started, and a little tact and sympathy should be shown by all connected in any way with hospital work.

COTTAGE NURSES.—A most interesting meeting will be held by the Hospitals Association at Westminster Hospital on January 15, at 8 p.m., when Miss Broadwood, of Ockley, will read a paper on "Cottage Nurses and Their Training," Mr. Henry C. Burdett in the chair. Miss Broadwood is well known for having established in many country districts in Surrey respectable women half-trained as nurses. The scheme seems to give great satisfaction and to work well; but the theory is one against which some of our best nurses are striving, and there is likely to be a lively discussion on Miss Broadwood's paper. Tickets can be had from the secretary of the Hospitals Association, at Norfolk House, W.C.

THE STRIKE AT LEITH.—We have several letters and newspapers sent us from Leith giving particulars as to the strike of nurses at the hospital there. There are, as usual, two sides to the question. One is that the nurses are overworked and have no time for recreation. The other is that the matron who has lately been appointed found matters in such an unsatisfactory state she had to adopt stringent measures to restore discipline. If the matron is in the right, it is a pity she permitted "Justice" to write the foolish letter which upholds her character by vilifying those of the doctors and nurses. As all the nurses have sent in their resignations the committee will doubtless make a rigid enquiry into the cause of such wholesale discontent.

PHILADELPHIA NEWS.—Our correspondent writes: "A meeting has been called to be held at the College of Physicians to endeavour to establish a Beneficial Fund for Nurses. The coming winter promises to be a busy one for both doctors and nurses, owing to the number of cases of 'typhoid fever' in the city and suburbs. Two of our most prominent young physicians are victims, and a petition signed by all the leading doctors has been sent to the Board of Health, asking for an enquiry into the continued pollution of the Schulkill river, and desiring them to direct all householders to boil the water before using for domestic purposes. You ask particulars of our different hospitals: they are very numerous, but I will mention one or two. The Philadelphia is the great hospital charity of the municipality. It is supported at an annual cost of about 140,000dols. The hospital will accommodate 1,200. The average daily number during the year 1888 was 1,144. All the patients are free. The Maternity Hospital, 734, South Tenth-street, is doing a useful work. It has twenty-two beds. During the year ending October 1, 1889, there were 153 patients received, of whom 84 were free. The cost per day for each person was 82 cents. There are no out-patients. The House of St. Michael and All Angels, Forty-third and Wallace-streets, where poor crippled coloured children find gentle friends, had 31 patients during the last hospital year. All are received free. The household expenses during the year, inclusive of wages, amounted to 1,039dols. There are no outward patients. The institution is supported entirely by voluntary contributions. It was started by Sister Sarah a little more than three years ago. She has had charge of it since. The oldest hospital in the country is in this city. It is the Pennsylvania Hospital, at Eighth and Pine-streets, which has been in successful operation for 137 years, and was never more flourishing than it is to-day. The earnings of the nurses connected with our Lying-in Charity reached over 5,000dols. last year.

### The Rights and Wrongs of Nursing Uniforms.

The late exhibition of nursing uniforms has caused an accession of interest on this subject, and many are the points with regard to what is best in the way of nursing costumes that we are called upon to decide. The general opinion is growing in favour of cotton frocks for the sisters, especially since the pretty uniforms of Charing Cross were invented. Miss Pringle, of St. Thomas's, put her sisters into cotton last summer, but only for the warm months. If a good, thick cotton is used, however—not cheap rubbish—there is no reason why cotton should not be worn all the year round. Blue is found to wash very well if it is not too dark, and the new deep red which Liberty introduced is a good wearing colour. With regard to caps, those tying under the chin, and those with long tails down the back, may be very dignified, but they are not comfortable; strings under the chin in hot weather add to the heat and seldom keep tidy. Very high caps with Cash's frilling neatly goffered are becoming, but the plain muslin triangular caps are most easily washed. Aprons should always be white; the scarlet ones at St. Saviour's are pretty, and the holland ones at St. Thomas's are ugly; but nothing looks so nice or wears so well as a white linen apron. There should always be two large pockets in every nursing apron, and the bib should be held up by straps crossing behind, as even safety pins ought not to be a necessity in a working woman's costume. Chatelaines are going out, because they jingle, and neat leather cases or sabretasches are worn, slung from the belt.

It is becoming more general for each hospital to give some sort of badge to the nurses who work within its walls. At Liverpool Royal the badge is a small silver star and bar worn on the left breast. At Birmingham General Hospital the nurses were last year all given a bronze star with the initials of the hospital engraved on it. The nurses at Charing Cross all wear the Maltese cross with *fleur de lis* between, known as the "Charing" cross; it is slung round the neck by a black and red ribbon. The nurses of St. John the Divine wear a bronze cross bearing the name of St. John, and slung round the neck by a bright blue ribbon. The Royal Red Cross is an order bestowed by the Queen on those Army or Navy nursing sisters whose services merit special distinction; it is a red Maltese cross worn on the left shoulder. Several of our nurses have also won the Egyptian star, bestowed by the Khedive, and a few have gained other foreign honours. The medal of the St. John's Ambulance Association sometimes worn by nurses, is a circle with the four ends of a cross sticking out; the name of the association is written round the circle.

With regard to outdoor uniforms we confess to being tired of the perpetual round black cloak and black bonnet and veil. At Liverpool Royal the cloaks are shaped and have a red hood; at Salisbury the nurses wear a violet veil; the private nurses of The London wear a green cloak and bonnet with white strings; at Charing Cross the cloaks are half-fitting blue cashmere with red lining; the St. John the Evangelist wear brown loaks, bonnets and veils; and the Army Sisters grey cloaks, bonnets and veils. But as a rule the outdoor uniform is black, with but slight variations; for instance, King's have long black veils, Guy's short veils, and Bart's no veils; the National and Metropolitan Association have a blue edge to the black ribbon on the bonnets. The lady pupils of King's College can be told by the grey gowns which show under the black cloaks, but they also wear black bonnets and veils. The Glasgow district nurse has a cape to her cloak, which is both sensible and becoming. Blue outdoor uniform is worn by the nurses of the Devonport Albert Hospital, Brighton County Hospital, Jersey Nurses' Institution, St. Saviour's Hospital, South London Association, and many more country and provincial institutions. There certainly seems no reason why a nurse's cloak should not be made half-fitting, and many neutral tints will be found to wear as well as black. Here is an opening for an enterprising manufacturing firm in co-operation with the dressmakers and matrons.

The necessity for all engaged in hospital work to wear washing materials is now recognised, but there are some woollen materials which wash excellently—for instance, the grey uniforms of the Army Sisters, and the brown religious dress of the East Grinstead Sisters. Still, the preference should be given to cotton materials, and every hospital should supply every woman within its walls with three washing dresses, six caps, and twelve aprons. The late exhibition of uniforms fully demonstrated that there is not the slightest difficulty in designing a nursing dress which should at once be useful and beautiful.

### Keeping Christmas.

Christmas Day found the managers of the **Dreadnought Seamen's Hospital at Greenwich** ready to pay ample respect to such a good old English custom. The larder sent forth mountains of provisions, there being over a score of turkeys, huge pieces of beef, and numerous puddings to feed the sailors representing no less than twenty different nations, the chief among them being English, Swedes, Norwegians, Scotch, French, Germans, Irish, Australians, Danish, Canadians, Belgians, Chilians, Cingalese, and other Asiatics. The dinner was served at 12.30 sharp, the officers undertaking the carving at the different tables. After dinner the men were served out an ounce of tobacco each and a pipe, and those who were well enough occupied themselves during the afternoon in singing songs, spinning yarns, and in the many other ways that "Jack" so easily finds for filling up his spare time. During dinner the wards were visited by members of the committee and other well wishers of the hospital.

On Christmas Eve the patients of **Devonshire Hospital, Buxton**, assembled in the men's dayroom, and appeared to thoroughly enjoy the festivities there provided for them. Songs and recitations were given by the nurses and patients, the former doing all in their power to make the poor sufferers happy. Presents, consisting of scarves, gloves, handkerchiefs, kneecaps, and other useful articles, were given to every patient. On Christmas Day the patients had their usual Christmas dinner and tea, and at the latter meal each wore the contents of "bon-bons" which were given to them, causing much amusement. Both dayrooms were tastefully decorated with holly, mottoes, etc., which gave a cheery aspect to them. A Christmas tree in the women's room was quite a novelty, being embellished with dolls, lighted candles, and sundry other articles. Much credit is due to the nurses for their zeal in promoting enjoyment for all.

Christmas Day was spent very happily at the **Darlington Hospital**. The wards had been prettily decorated, and liberal supplies of provisions had been sent for the Christmas fare. The dinner consisted of turkeys, plum pudding, and custard, with lemonade to drink. Afterwards dessert was arranged on the tables, so that the patients might offer it to their friends who came to spend an hour with them during the afternoon. A short bright service was held in the men's ward by Arthur Pease, Esq. All the patients who were able were present, and the household also. In the evening an entertainment was given by the officials. The dispenser, acting as Quack Doctor, was introduced to the patients, and was followed by the porter, dressed as Old Father Christmas, wheeling in the "doctor's cart," which consisted of a large linen basket covered with red and hung round with bottles, the whole being placed on the stand of the ambulance and lighted with Chinese lanterns. After an amusing speech from the Quack Doctor, interrupted by much laughter, a few parcels were distributed to the men which contained toys that had been specially addressed to them. The fun was fully appreciated, and one old man who had unfortunately been omitted showed considerable disappointment. The basket was then opened and was found to contain a quantity of useful presents which gave great pleasure to the recipients.

At the **Infirmary, Cardiff,** on December 24, the distribution of presents in the children's ward from the Christmas tree passed off admirably. The wards were all elaborately decorated and illuminated with coloured globes. On Christmas Day the patients had the time-honoured roast beef and the prehistoric plum pudding. The hospital air in the male wards was for once rendered smoky by those patients who believe in the therapeutic value of the nicotine plant. The day was wound up with a little music in the women's ward. The festivities were finished on New Year's night with a concert, which all the patients who were able attended, and afterwards the nurses had a small dance. A few of the nurses presented the house surgeon, Dr. Thomas, with a handsome pair of bronze statuettes "as a slight token of gratitude for his kindness during his course of lectures to them."

At **Congleton Cottage Hospital,** through the kindness of several friends, Miss Wagnell, the matron, was enabled to give the inmates, a large number of old patients, and about a hundred poor children, a very substantial tea. After tea the secretary of the hospital distributed a large number of useful garments to the patients, and fruit, sweets, and toys to the children. Several ladies and gentlemen contributed to the enjoyment of the evening by songs, etc.

**Nottingham General Hospital** had more decorations and more visitors than usual. Miss Fox presented Division 6 with a splendid musical box, as a sort of reward for the attention lavished on Judge Bristowe. The judge himself took the greatest interest in the Christmas festivities. Many of the patients declared that they had never spent such a pleasant Christmas Day before. The annual Christmas tree was held on January 4, and the nurses' dance will take place on January 22.

Christmas at the **Victoria Park Hospital** was in every way a bright and happy one, everything that could be done to make the day a happy one and remove for a time the thought that we were in a hospital. The beautiful corridors had quite a festive look decorated with holly, flags, and Chinese lanterns. All patients who were able to leave their wards were, through the kindness of the house physician, allowed to be up and walking about. At 11 a.m. there was a service in the hospital chapel, which all who were able attended. After service a real good old English dinner was served of roast beef, plum pudding, and fruit. The tables were beautiful with plants and evergreens. After dinner the men had what is dear to the heart of every man, a good smoke, and the women a refreshing cup of tea. In the evening a concert was given by ladies and gentlemen, friends of the matrons, who deserve great praise, who, leaving their own family circle, came to brighten for an hour or two the suffering ones. After singing the National Anthem coffee was served, and so finished Christmas Day of 1889. The young patients all received very pretty presents, some boxes of games, and others books. On Boxing Day another concert was given, including some *tableaux vivants*. "Where are you going to, my pretty maid?" was sung with great taste by one of the patients. Several selections on the piano were given by one of the resident doctors; songs were also sung by nurses and patients.

The dinner of the patients at the **Carmarthenshire Infirmary** on Christmas Day was of a most sumptuous description. It included goose, turkey, plum pudding, and dessert. The matron (Miss Macintyre) and her assistants were most kind to one and all. The children's ward had quite a festive appearance, and their hearts were greatly gladdened with the cards and other things distributed amongst them. An entertainment was held on the 8th.

The acting at the **Great Ormond-street Children's Hospital** entertainment, when "Raising the Wind" was played, was so much above the average it deserves special mention. Jeremy Diddler was excellent, acted by Mr. J. Valérie, and Miss Philippa Hicks, as Laurelia Durable, an amorous old maid, was simply killing. The evening was a very merry one.

**Wallasey Cottage Hospital** kept Christmas on the 26th. The interior had been beautifully decorated with evergreens, and in the hall were rows of coloured lamps, the effect being decidedly picturesque. This was the work of Miss Garvin, the hard-working matron, and a few friends. At the base of the stairs was a Christmas tree, brilliantly lit up, and laden with presents of all kinds, suitable for old and young. In one of the front wards about sixty—including not only the present inmates, but those who had been inmates at any time during the year—sat down to a substantial tea. Afterwards there were presents, some acting, and some songs.

At **Salford Royal Hospital** the Mayor distributed the presents to patients, nurses, and the household staff. A beautiful fir tree had been placed in the Boutflower Ward, the top of which nearly touched the ceiling. This was laden with 140 presents, chiefly articles of clothing. The branches were also illuminated with coloured wax lights and tiny lanterns. Boxes of chocolate and bon-bons were also numerous. From the ceiling depended festoons of evergreens.

Santa Claus visited the **Jenny Lind** on New Year's Eve. Dressed in a long gorgeous robe and with a flowing white beard, Mr. Harcourt made a famous Father Christmas. Having made a low obeisance to the Mayor and Mayoress of Norwich and saluted the company generally, he proceeded, with the assistance of his "wife," impersonated by Mr. Cecil Barwell, to distribute the contents of his "tree." A magic lantern was shown early in the evening, and the wards had been decorated by Miss Wainwright and her nurses.

New Year's Day was kept at **St. Thomas's Hospital.** The Victoria Ward was gaily decorated for the occasion, and a large Christmas tree adorned the centre table. It was 10ft. in height, and its branches were literally covered with toys. At four o'clock the tree was illuminated with coloured lamps, and the Victoria Ward children who could not leave their cots were all dressed in blue flannel jackets, presented to them by Mrs. Stone, wife of the treasurer, Alderman Stone. Upwards of sixty children sat down to tea.

At **Huddersfield Infirmary** No. 1 (Accident) Ward had been effectively decorated by the nurses and others, with the assistance of such of the patients as were able to render it, and hither all the inmates of the institution, with the exception of those whose condition did not permit of their removal, had been brought to enjoy the entertainment provided for them. After the distribution of presents Mr. Summers made a short speech, and then there were some merry songs and an operetta.

**Croydon Hospital,** under the direction of the matron and house surgeon, was completely transformed for the festive season, the various wards being plentifully and artistically decorated with evergreens. At mid-day a good substantial dinner was placed before the more convalescent patients, which was thoroughly enjoyed, after which the afternoon was given over to various amusements, of which music and singing formed the principal items. In the evening several of the nurses dressed themselves in fancy costumes representing various characters, and visited the wards.

At the **Evelina Hospital** on Tuesday last there were grand doings, and the Lord Mayor attended in state in his coach and four. Furtive little faces glanced timidly at the man in the gorgeous scarlet robes with a diamond badge glittering on his breast, and they were also much impressed by the sheriff. The wards were profusely decorated and hung with flags and lanterns, and in each was a huge Christmas tree covered with pretty presents. The children all had buns for tea, and generally enjoyed themselves.

At **Sheffield Infirmary** on New Year's Eve there was a Christmas tree for fifty little patients who were within its walls. Lots of visitors gathered to help distribute the toys, and the house surgeons, matron, and nurses all did their best to make the evening pass off pleasantly.

### Everybody's Opinion.

*[Correspondence on all subjects is invited, but we cannot in any way be responsible for the opinions expressed by our correspondents. No communications can be entertained if the name and address of the correspondent is not given, or unless one side of the paper only be written on.]*

### OVERWORKED NURSES: A CORRECTION.

MISS PRETTY, matron of the Miller Hospital, Greenwich, writes: In this week's number of THE HOSPITAL "E. C." mentions the Miller Hospital as one of the institutions in which the nurses, every other day, are on duty for fourteen hours. This statement is quite without foundation; in proof of which I will tell you what our nurses' "off-duty" time really is. Staff nurses, every other Sunday, 2 to 10 p.m.; every weekday except Thursday, two hours, either in the afternoon or evening; once a month "day off," *i. e.*, they do not come into the ward at all. Probationer, every Sunday, 10 to 1.30, every weekday, 2 to 4 p.m., once a week, 2 to 10 p.m. It occasionally happens that the nurses have to wait five weeks for their "day off," but in this case it is always made up to them when the work is slack, and in no case within my recollection have they been deprived of this privilege. Also as regards the daily two hours. When the wards are very heavy I have often to insist on my nurses going off duty, and if I have occasion to ask them to stay on, they do so without grudging. I venture to add that no one worthy of the name of a nurse would object to staying on duty for an extra hour occasionally should circumstances call for it. I think this time table will compare favourably with that of other hospitals.

### Presentation.

ON Tuesday at Guy's Hospital Mrs. Lushington presented to numerous nurses the Butterworth medals, lately instituted as a reward for long and meritorious service by Mr. J. W. Butterworth. The medal is of silver and bears the head of Thomas Guy, founder of the hospital, on one side, and the name of the recipient nurse on the other. It is to be given in future to every nurse who serves the hospital satisfactorily for five years. On Tuesday it was presented to the matron, Miss Jones, and to Sisters Patience, Clinical, Grace Cornelius, and the Home Sister; also to Nurses Newman, Margaret Kehall, Butler, Phillips, Steels, Rawlinson, Hardy, Male, Woolcraft, Peckham, Alston, Simms, and Whiskey. Some songs and recitations were given when the presentations were over.

### Appointments.

KIMBERLEY HOSPITAL.—Nurse Bayes has been appointed outside midwife to this hospital in South Africa, and sails this month for her new home. Nurse Bayes trained at the Rotunda, and holds excellent certificates; she has lately been working at Norwich. We wish her every happiness in return for her courage in thus venturing so far afield.

### Notice.

THE Exhibition of Nursing Uniforms will be on view at the Hahnemann Hospital, Liverpool, on January 13, 14, and 15, from 10 till 2 in the morning, and from 5 to 8 in the evening. Admission, 1s.; nurses in uniform free.

### Notes and Queries.

### Queries.

(37) *Lectures.*—Where can I get lectures on nursing, and the loan of medical books for a small fee? *Nurse Hare.*

(38) *St. Barnabas Guild.*—To whom should I apply to become a member of the Guild of St. Barnabas. What is the fee? *Adjutant.*

(39) *Bolton Scandal.*—In what hospital did the late Nurse Morris train? *Bournemouth.*

(40) *Books on Nursing.*—Which is the best book on district nursing, and the best on medical and surgical nursing? *Vera.*

### Answers.

(37) *Lectures.*—At the Midwives Institute and Trained Nurses' Club, 15, Buckingham-street, Strand. Write to the secretary for particulars.

(38) *St. Barnabas Guild.*—Apply to the secretary, 26, Brooke-street, Holborn.

(40) *Books on Nursing.*—"A Guide to District Nurses," by Mrs. Dacre Craven, price 2s. 6d.; "Lectures to Nurses," by Miss Luckes, price 2s. 6d.

### Vacancies.

### Matron.

Wigan Infirmary (assistant), £40.

### Sister.

London Fever Hospital, £30.

### Night Superintendent.

North Staffordshire Infirmary, £30.

### Nurses.

Bath United Hospital (several), £25.
Blackheath Institution.
Bournemouth Institution, £25.
Cambridge Nurses' Home.
Canterbury Hospital, £25.
Cardiff Infirmary.
Chelsea Hospital for Women, £25.
Chelsea Infirmary, £18.
Cheltenham Institution.
Clapham Association.
Croydon Hospital (two).
Darlington Hospital.
Devonport Albert Hospital, £25.
General Nursing Institution.
Grimsby Nursing Home (two).
Hitchin Nursing Institution, £25.
H. M. Hospital, Stepney, £25.
Kimberley Hospital, South Africa.
Kidderminster Children's Hospital.
Liverpool Infectious Hospital (four), £25.
Newcastle Nurses' Home.
Newark Hospital, £25.
Nice and Mentone, £3 a month.
Norwich Nurses' Home.
Nursing Institution, 96, 97, 98, Wimpole-street, London, W., Mr. Wilson has several vacancies for thoroughly-trained nurses.
Oxford Military College, £30.
Peterborough Infirmary (night), £25.
Richmond Union (two).
Rochdale Infirmary, £22.
Scarborough Institute.
South London Institute (several).
St. George's Convalescent Home.
St. Leonards Convalescent Home.
St. Nicholas Home, £20.
St. Pancras Union (two), £20.
St. Saviour's Infirmary, £20.
Ventnor Consumption Hospital, £25.
Weston-super-Mare Hospital, £25.
Windsor Infirmary (night), £20.
Workhouse Infirmary Association.

### District Nurses.

Fowey, Cornwall.
Halifax.

### Probationers.

Birmingham Children's Hospital.
Boston Hospital.
Haywood Hospital.
Hertford Infirmary.
Hull Royal Infirmary.
Kilmarnock Infirmary.
Mr. Walter's Hospital, Manchester.
Newport Infirmary.
Torbay Hospital.
Workhouse Infirmary Nursing Association.

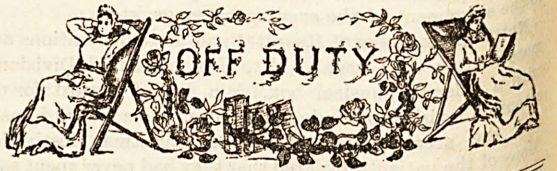

### N.B. FOURTH QUARTERLY WORD COMPETITION

### Commenced Jan. 4th, 1890, ends March 29th, 1890.

Three Prizes of 15s., 10s., 5s., will be given for the largest number of words derived from the words set for dissection.

Proper names, abbreviations, foreign words, words of less than four letters, and repetitions are barred; plurals, and past and present participles of verbs, are allowed. Nuttall's *Standard* dictionary only to be used.

N.B.—Word dissections must be sent in WEEKLY, arranged alphabetically, with correct total affixed.

The word for dissection for this, the SECOND week of the quarter, being "PANTOMIME."

### Results of Thirteenth Week.

| Names. | Jan. 2nd. | Totals. |
|---|---|---|
| Winifred | — | 672 |
| Adelaide | — | 646 |
| Nettie Vance | — | 757 |
| F. W. P. H. | — | 1673 |
| Amos | — | 596 |
| Reyot | — | 369 |
| Ellice | 144 | 1820 |
| A. S. K. | — | 275 |
| Neptune | — | 1314 |
| Ecila | 181 | 1942 |
| Tinie | — | 17 |
| Amicus | — | 946 |
| Juvenis | — | 539 |
| Jumps | 192 | 1940 |
| Sister | — | 438 |
| M. W. | — | 1684 |
| Esperance | — | 1683 |
| Sunshine | 149 | 1605 |
| Electrician | — | 1593 |
| H. C. | — | 662 |
| Reldas | 161 | 1814 |
| Rover | — | 776 |
| Qu'appelle | 138 | 1499 |
| Beryl | — | 188 |
| Lightowlers | 198 | 1869 |
| Jenny Wren | 168 | 1404 |
| Hercules | — | 12 |
| Night Watch | — | 346 |
| Oakenrod | — | 1170 |
| Embryo | — | 385 |
| Gypsye | — | 967 |
| Leo | 94 | 938 |
| Pearl | — | 299 |
| Africando | — | 171 |
| Lady Clara | — | 133 |

### RESULTS OF THIRD QUARTERLY WORD COMPETITION.

*First Prize* (15s.) is awarded to "Ecila" (Miss E. A. Todd, Moulsae, Newport Pagnell.)

*Second Prize* (10s.) is awarded to "Jumps" (Mr. Thomas A. McIntyre, 86, Grosvenor-road, S.W.)

*Third Prize* (5s.) is awarded to "Lightowlers" (Miss F. C. Chadwick 2, Canning-street, Liverpool.)

### Notice to Correspondents.

*Lightowlers.*—Totals, *twelfth week*, for 1671 read 1711.

